# Nasal Dermoplasty for Recurrent Polyps in a Patient with Churg-Strauss Syndrome

**DOI:** 10.1155/2015/192804

**Published:** 2015-01-27

**Authors:** George Anastasopoulos, Theophanis Machas, Sofia Kegioglou, Demetra Rapti

**Affiliations:** ^1^Department of Otolaryngology, Hygeia Hospital, 4 Erythrou Stavrou Street and Kifisias Avenue, Marousi, 151 23 Athens, Greece; ^2^Department of Anaesthesiology, Hygeia Hospital, 4 Erythrou Stavrou Street and Kifisias Avenue, Marousi, 151 23 Athens, Greece

## Abstract

Nasal dermoplasty for recurrent polyps (NDRP) is a new technique for the surgical treatment of this condition. Churg-Strauss syndrome is characterized by the presence of nasal polyps with a great tendency for relapse after surgical or pharmaceutical treatment. It is the first time that we use NDRP to treat nasal polyps in a patient with Churg-Strauss syndrome. The patient was a 33-year-old female suffering from Churg-Strauss syndrome having had multiple operations in the past for recurrent polyps. NDRP was performed on the left nostril only. The mucosa of the left nasal vault was replaced by a split-thickness skin graft (modified dermoplasty). On the right nostril, polyps were removed and the ostia of the paranasal sinuses were enlarged as in typical endoscopic sinus surgery. The skin graft had a successful take and eight months after the operation no polyps are detected on the left side while polyps have recurred on the right nasal cavity. Applying the technique to a single nostril has several advantages, namely, the reduction of the operational time and therefore the risks for the patient from a prolonged general anaesthetic and the ability to judge the efficacy of the technique on the same patient.

## 1. Introduction

On April 2013, we first published our modified nasal dermoplasty technique for the treatment of recurrent polyposis (NDRP) [1]. The rationale for the technique is the replacement of the mucosa of the ethmoidal vault, responsible for the generation of polyps, by a split-thickness skin graft. So far our results have been promising showing high percentage of graft take and complete cessation of polyp formation.

In this paper we present the case of a patient suffering from Churg-Strauss syndrome, a condition characterised by its tendency for recurrent nasal polyp formation despite radical removal [2].

The patient had polyps removed from both nostrils but NDRP performed only on her left side. Thus, we evaluated the efficacy of the technique on the same patient under the same environmental conditions and medication.

We report on the efficacy of NDRP technique in controlling the relapse of nasal polyps. We also discuss one of the major concerns of this technique which is the foul odor due to the infection of the graft's keratin debris and we present certain modifications of our original technique.

## 2. Case Presentation

A 33-year-old female patient suffering from Churg-Strauss syndrome presented with massive nasal polyposis and a history of three previous attempts of removal by means of endoscopic sinus surgery. The patient was informed about the potential benefits of NDRP and consented to the procedure.

The patient was given a preoperative regimen of amoxicillin/clavulanic acid and methylprednisolone and then a CT scan was performed. NDRP was performed on the left nostril which was more affected. In the right nostril, polyps were removed and the ostia of the sinuses were widened as needed.

In the left nostril, polyps were also removed, the previously created antrostomy was enlarged, and most of the anterior wall of the sphenoid sinus was removed to facilitate the insertion of the skin graft. The bony septa of the ethmoid cells hanging from the nasal vault and inserted into the lamina papyracea were then removed, using a 15° diamond burr to avoid the tent effect when applying the skin graft.

The final step of the preparation of the surgical cavity was to meticulously remove any small remnants of nasal mucosa in the nasal vault, the lamina papyracea, and the lateral surface of a small remnant of the middle turbinate that was present due to the previous operations and was preserved. The rational was to avoid the formation of a mucocele postoperatively.

A 10 × 7 cm, 0,15 mm thick, skin graft was taken from the inner surface of the left thigh. Two longitudinal midline incisions on both sides and multiple small incisions on the surface of the graft were performed.

The graft was inserted into the left nostril covering the lamina papyracea, the fovea ethmoidalis, and the upper 1 cm of the septum transversely, from the posterior limit of the frontal ostium to the area corresponding to the sella turcica longitudinally. A meticulous effort was made to firmly apply the graft against the above mentioned surfaces, removing air between the graft and the surgical cavity. No nasal packing was used.

The procedure was completed within five hours and the patient left the hospital the next day. She received amoxicillin/clavulanic acid 1 gr twice daily for one week and was instructed to rinse both nostrils with saline as often as possible for a month.

**Figure 1:**
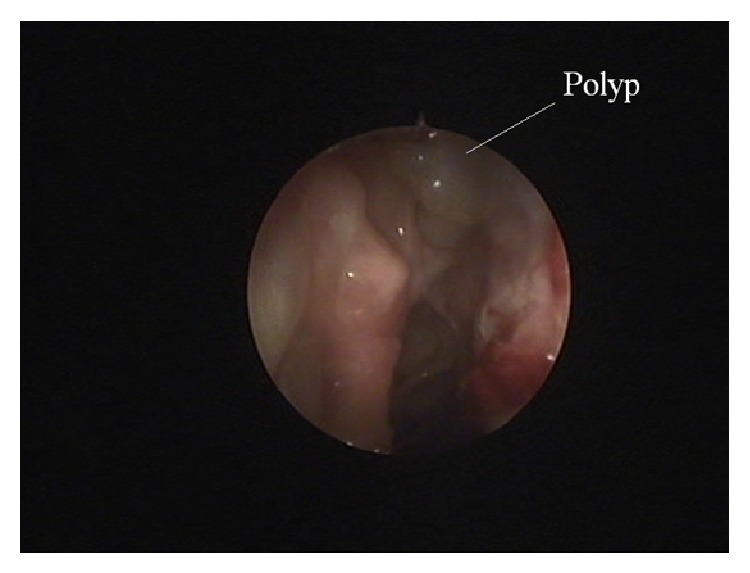
Right nostril. Day 8. Early eruption of polyps.

The patient had a normal postoperative course complaining only about a minor disturbance on the thigh area. Starting from postoperative day 8, the patient was examined endoscopically at various intervals. On day 8 the first sign of polyp formation was detected on the right side (Figure 1). The graft on the left side appeared viable in between areas covered by desiccated blood clots, despite signs of necrosis at the periphery.

**Figure 2:**
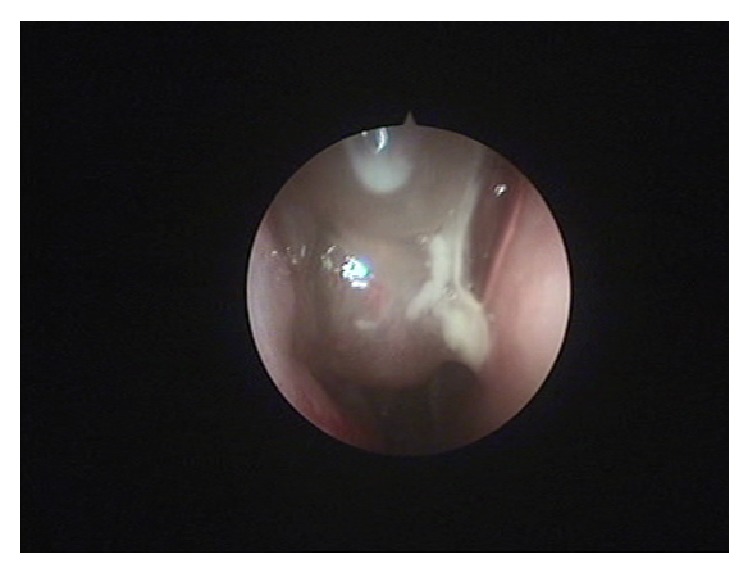
Right nostril. Polyp relapse.

In the months that followed, prominent polyp formation was noted on the right side (Figure 2) with signs of size fluctuation mostly due to environmental conditions as the patient did not receive medication on a regular basis.

**Figure 3:**
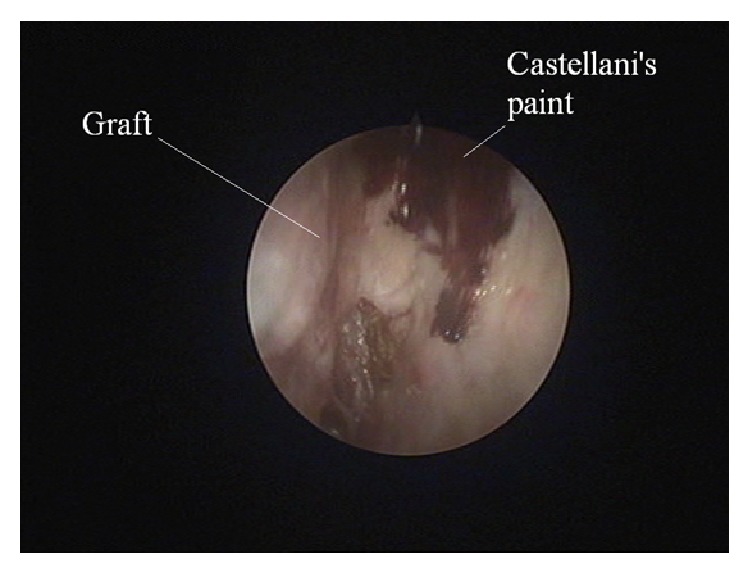
The graft eight months after the operation. The nostril is free of polyps and no infected debris is observed.

Eight months after the operation, no polyps have developed on the left side (Figure 3), except mild polypoid degeneration of the septal mucosa at a small slit-like area between the slightly deviated septum and the area corresponding to the removed agger nasi. The graft appeared clearer at every visit making it easier to assess its viability.

**Figure 4:**
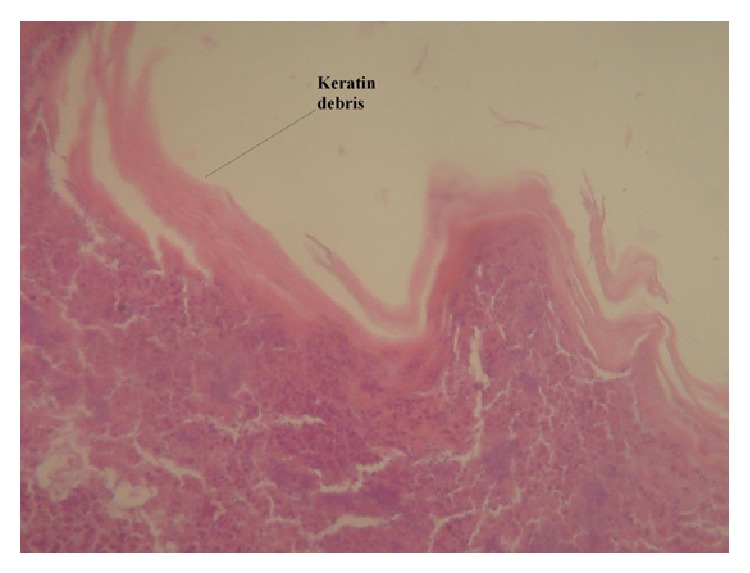
Biopsy specimen: horny keratin layer of epidermal type with underlying chronic and acute inflammatory cells. Magnification: ×100, tissue stain: hematoxylin-eosin.

One month after the operation, the patient started complaining about a foul odor, at times detected also by her relatives. The cause of the fetor was infection of the keratin debris of the graft by the normal nasal flora. The presence of the debris was confirmed by biopsy (Figure 4).* Klebsiella* spp. was isolated by culture, sensitive to most common antibiotics.

To treat fetor, several measures were used including saline nasal rinses, removal of debris at the surgery, oral antibiotics, and locally applied Castellani's paint on the skin graft with a malleable cotton applicator. We noted that the most effective and less discomfort producing measures were the intranasal use of tobramycin drops three times daily for several days to treat the infection and a nasal ointment containing lanolin and paraffin to help removal of crusts.

Eight months after the operation, the patient is completely polyp-free on the dermoplasty side. She reports an improvement in the respiratory function in general, with improved nasal breathing even through the right nostril which was operated using the conventional endoscopic technique when compared to previous interventions, with only sporadic bouts of foul odor, especially on warm days with increased environmental humidity.

## 3. Discussion

A 33-year-old female patient suffering from Churg-Strauss syndrome underwent our modified nasal dermoplasty for recurrent polyps (NDRP) [1]. The operation was successful and the benefit from dermoplasty was significant. Eight months after the operation, the patient has no polyps on the left nostril while on the right side the formation of polyps begun as early as the eighth postoperative day. Also, dermoplasty seems to have a beneficial effect on the other nostril, as the patient reports that both nostrils remain functional with little or no medication especially when compared to her condition after a similar amount of time following her previous operations.

The main complaint of the patient was an intense foul odor coming from the dermoplasty side at times perceptible by other people as well. The cause of the odor is the infection of keratin debris of the graft by the normal nasal flora, a condition commonly observed in cases of intranasally used skin grafts [3–6]. Our recommendations for the management of this condition, based on our experience as well as on previous reports, include the removal of crusts under endoscopic control, the intranasal administration of tobramycin eye drops [7] to treat the infected debris, and the intranasal administration of oily ointments to help detach and remove the crusts. The frequency of the application of these measures has yet to be determined but certainly depends on clinical response. The use of oral antibiotics was not particularly effective, and the locally applied Castellani's paint [8] on the skin graft, though effective, added to the patient's discomfort.

This case had several unique characteristics compared to our previously published series. The technique was applied to a single nostril, thus significantly reducing operational time and the risks of prolonged general anaesthesia for the patient. This also allowed us to compare the efficacy of dermoplasty versus typical FESS in the same patient under the same medication and environmental conditions.

The skin graft was significantly larger compared to our previous cases and was processed thoroughly performing multiple small incisions on its surface. This way, air and blood are prevented from accumulating under the graft facilitating its take. Moreover, small mucosa remnants may find their way to the surface preventing the formation of mucocele. In addition, the graft was cut longitudinally from both sides leaving a small bridge of skin in the midline. This results in more areas of graft regeneration reducing the amount of shrinkage.

If the middle turbinate is intact, it must be stitched to the septum using a long-term absorbable suture. If, as in the case presented here, only a small remnant of the middle turbinate is present, this remnant must be preserved but the graft may have to cover the upper surface of the septum as well. In this case, care is needed when detaching the mucosa from the cribriform plate that bears the endings of the olfactory filaments to avoid cerebrospinal fluid rhinorrhea. The mucosa of the cribriform plate must be removed with a small size microdebrider. The removal of the middle turbinate must be avoided as it may produce changes leading in atrophic rhinitis [9]. No signs of nasal mucosa atrophy or viscous nasal discharge suggestive of atrophic rhinitis were observed in this case.

Polyps have to be removed from both nostrils. Regarding the side, more appropriate for the performance of NDRP we think that it is better to perform dermoplasty on the most convenient side first, for example, the side at which the septum is less deviated. The reason for this is that, after several months, if the patient wishes to have dermoplasty performed on the other nostril as well, it will not probably be necessary to remove polyps from the already operated side, giving time to correct any septal deviation.

Finally, the formation of a well-defined cavity at an earlier stage was advantageous in this case, as only the mucosa had to be removed and the skin graft inserted.

## 4. Conclusions

NDRP is an effective technique to treat nasal polyps even in patients with established tendency for polyp formation. Certain modifications of the original report are presented, regarding the single-side operation, the amount of graft needed, and the postoperative management. The main complication is a foul odor due to infection of the graft's keratin debris. This particular complication is well known from other cases of intranasal use of skin grafts and is treated conservatively. To our knowledge NDRP is the only technique that can claim the prevention of nasal polyp relapse.
